# Warm Autoimmune Hemolytic Anemia Associated With Asymptomatic SARS-CoV-2 Infection

**DOI:** 10.7759/cureus.14101

**Published:** 2021-03-25

**Authors:** Joseph R Liput, Kim Jordan, Rini Patadia, Elizabeth Kander

**Affiliations:** 1 Internal Medicine, OhioHealth Riverside Methodist Hospital, Columbus, USA; 2 Hematology/Oncology, Columbus Oncology Associates, Columbus, USA

**Keywords:** severe acute respiratory syndrome coronavirus 2 (sars-cov-2), warm autoimmune hemolytic anemia, coronavirus disease 2019 (covid-19), anemia

## Abstract

Severe acute respiratory syndrome coronavirus 2 (SARS-CoV-2) and the resultant coronavirus disease 2019 (COVID-19) are associated with several hematologic abnormalities, including immune thrombocytopenia, antiphospholipid syndrome, and autoimmune hemolytic anemia (AIHA). Initial case reports suggested immune dysregulation to be the underlying etiology of SARS-CoV-2-associated AIHA, as all reported cases involved patients with moderate to severe COVID-19, many of whom had underlying lymphoproliferative disorders. More recently, AIHA has been reported in patients with mildly symptomatic SARS-CoV-2 infection. Here, we detail a patient with asymptomatic SARS-CoV-2 infection who presented with severe, symptomatic anemia. Workup was consistent with warm autoimmune hemolytic anemia (WAIHA) secondary to SARS-CoV-2 infection.

## Introduction

Autoimmune hemolytic anemia (AIHA) is an acquired hemolytic disease defined by the presence of anti-erythrocyte antibodies. There are several known secondary causes of AIHA, the most common of which are B-cell malignancies, rheumatologic diseases, and medications [1]. Though rare, viral infections were known to be a secondary cause of AIHA prior to the SARS-CoV-2 pandemic [2]. Through the publication of several case reports, SARS-CoV-2 infection is now established as a secondary cause of AIHA. The following case adds to the growing literature detailing the association between COVID-19 and AIHA and is made unique by the patient’s asymptomatic infection with SARS-CoV-2. 

## Case presentation

A 33-year-old female with a history of iron deficiency anemia presented to the emergency department with a three-day history of dizziness and tinnitus. On initial evaluation, vital signs were normal except for tachycardia of 111 beats per minute. Physical examination was notable for scleral icterus and jaundice. Complete blood count was significant for macrocytic anemia with a hemoglobin (Hgb) level of 6.5 g/dL (13.3 g/dL 1.5 years prior) and mean corpuscular volume (MCV) of 106.9 fL; platelet and white blood cell count were normal without lymphopenia. The hepatic function panel was significant for indirect hyperbilirubinemia (3.0 mg/dL) with a total bilirubin of 3.5 mg/dL. The metabolic panel was otherwise unremarkable. SARS-CoV-2 reverse transcription-polymerase chain reaction (RT-PCR) testing obtained from the nasopharynx prior to admission was positive. The patient had been exposed to SARS-CoV-2 10 days prior to admission when her son was diagnosed with COVID-19. She denied symptoms consistent with COVID-19 and a single-view chest X-ray was without infiltrates. Inflammatory markers including C-reactive protein (CRP), D-dimer, and fibrinogen were also normal.

**Figure 1:**
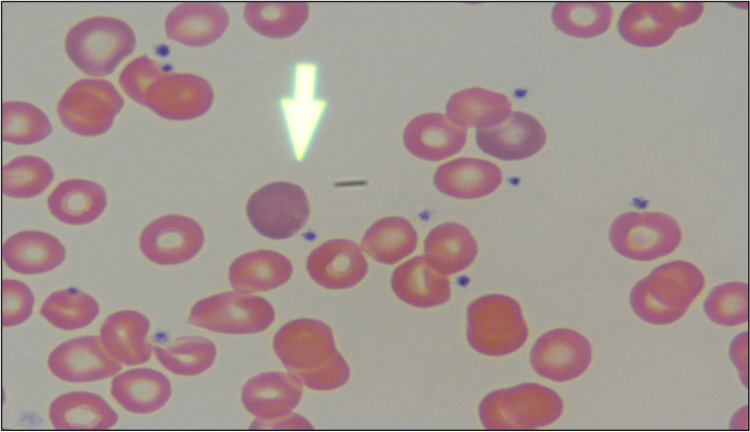
Blood smear demonstrating increased polychromasia (arrow: polychromatophilic red blood cell)

Further evaluation revealed an elevated lactate dehydrogenase (LDH) of 344 U/L (reference range 100-250 U/L) and undetectable haptoglobin (< 10 mg/dL). Reticulocyte percentage was 11.8% with a reticulocyte index of 3.0. Peripheral blood smear demonstrated macrocytic anemia with anisocytosis and mild polychromasia without schistocytes (Figure 1). 

Direct antiglobulin test (DAT) was positive for immunoglobulin G (IgG) and C3 and warm autoantibodies were isolated by elution. CT scans of the chest, abdomen, and pelvis were negative for lymphadenopathy, hepatosplenomegaly, or pulmonary infiltrates. Antinuclear antibody (ANA), HIV antibody, hepatitis C virus antibody, and hepatitis B virus surface antigen, surface antibody, and total core antibody were negative. A diagnosis of warm autoimmune hemolytic anemia presumed secondary to SARS-CoV-2 infection was made and the patient was initiated on 1 mg/kg/day of prednisone. She received one unit of packed red blood cells for hemoglobin of 6.2 g/dL with an adequate response. By day 6 of admission, hemoglobin had stabilized at 8.1 g/dL, LDH was within normal limits (209 U/L), and total bilirubin had normalized (1.3 mg/dL). The patient’s tinnitus and dizziness had resolved. She was discharged with 1 mg/kg/day prednisone. Follow-up laboratories six weeks after discharge demonstrated complete hematologic response with Hgb 12.6 g/dL and normal LDH and haptoglobin. The patient was continued on a prednisone taper.

## Discussion

Autoimmune hemolytic anemia is a rare condition affecting approximately one to three per 100,000 people per year; warm autoimmune hemolytic anemia (WAIHA) accounts for 65% of all cases [3]. Secondary causes for WAIHA are identified in 50% of cases and most commonly include B-cell hematologic malignancies, rheumatologic diseases, infections, and medications [1]. Since the start of the SARS-CoV-2 pandemic, cold agglutinin disease (CAD) and WAIHA have been diagnosed in several patients with COVID-19 [4-8]. The largest case series to date featured seven patients who were diagnosed with AIHA following SARS-CoV-2 infection, four of whom had WAIHA. Of the four patients with WAIHA, two patients had chronic lymphocytic leukemia (CLL) and one patient had monoclonal gammopathy of undetermined significance (MGUS) [5]. The fourth patient did not have any secondary pathology, but unlike our patient, had severe COVID-19. All seven patients had evidence of COVID-19 pneumonia and had elevated markers of inflammation (fibrinogen, D-dimer, CRP), and three patients required ICU-level care [5].

It is thought that many of the deleterious effects of COVID-19 stem from dysregulated immune response, which is further supported by randomized trials demonstrating mortality benefit with dexamethasone in patients with moderate to severe disease [9,10]. Thus, a dysregulated immune response could lead to the production of autoantibodies to erythrocytes, leading to AIHA in the setting of COVID-19. This hypothesis was initially supported by the fact that the first published cases of SARS-CoV-2-associated AIHA featured patients with symptomatic SARS-CoV-2 infection, many of whom had severe COVID-19. 

Our case is unusual in that our patient had no respiratory symptoms to suggest COVID-19 and her chest X-ray and chest CT were negative for pneumonia. She was maintained on ambient air throughout the hospitalization. Markers of inflammation including fibrinogen, D-dimer, and CRP were all normal and evaluation for more common causes of AIHA including lymphoproliferative disease, autoimmune disease, and other viral infections, was negative. The patient was taking ibuprofen daily prior to admission, which was discontinued while she was hospitalized. Haptoglobin remained undetectable four weeks following discontinuation of ibuprofen making drug-induced AIHA less likely. The timing of presentation ten days following SARS-CoV-2 exposure makes SARS-CoV-2-associated AIHA the most likely diagnosis. Our patient had a complete response with glucocorticoids. Little is known regarding the optimal treatment strategy of SARS-CoV-2-associated AIHA and follow-up data was limited in the largest case series to date [5]. More complex decision-making will arise in patients with severe hemolysis unresponsive to glucocorticoids, as second-line rituximab has the potential to impair humoral immunity in patients with active infection [11].

## Conclusions

This case adds to the growing body of literature demonstrating the connection between SARS-CoV-2 infection and AIHA. While initially thought to be secondary to immune dysregulation, our case demonstrates that SARS-CoV-2 may incite AIHA through other mechanisms, including molecular mimicry. SARS-CoV-2 infection should be considered in any patient presenting with new-onset AIHA of unclear etiology.
